# Invasive and Non-invasive Clinical *Haemophilus influenzae* Type A Isolates Activate Differentiated HL-60 Cells *In Vitro*

**DOI:** 10.20411/pai.v9i1.659

**Published:** 2024-04-17

**Authors:** Courtney L. Ferris, Marina Ulanova

**Affiliations:** 1 Department of Biology, Lakehead University, Thunder Bay, ON, Canada; 2 Northern Ontario School of Medicine University, Thunder Bay, ON, Canada

**Keywords:** Haemophilus influenzae, neutrophils, opsonization, innate immune response, HL-60 cells

## Abstract

**Background:**

The effective elimination of encapsulated bacteria like *Haemophilus influenzae* type a (Hia) relies on immune mechanisms such as complement-mediated opsonophagocytosis by neutrophils in coordination with opsonization by anti-capsular antibodies. This study evaluated if Hia could activate the immune response through neutrophils and if these responses differed between encapsulated versus unencapsulated or invasive versus non-invasive strains.

**Methods:**

HL-60-derived neutrophil-like cells (dHL-60), differentiated with 1.25% dimethyl sulfoxide over 9 days, were used in an opsonophagocytosis assay and *in vitro* infection model to measure Hia's susceptibility to killing and dHL-60 surface molecule expression, respectively. The impact of strain-specific features on the immune response was investigated using clinical isolates of a dominant North American sequence type (ST)-23, including Hia 11-139 (encapsulated, invasive), 14-61 (encapsulated, non-invasive), 13-0074 (unencapsulated, invasive), as well as a representative ST-4 isolate (Hia 13-240, encapsulated, invasive), and a nontypeable strain (NTHi 375, unencapsulated, non-invasive).

**Results:**

Unencapsulated and non-invasive Hi strains were more susceptible to killing by the innate immune response while the ST-23 invasive strain, Hia 11-139 required serum antibodies for destruction. Flow cytometry analysis showed increased expression of co-stimulatory molecule ICAM-1 and Fc receptors (CD89, CD64) but decreased expression of the Fc receptor CD16, revealing potential mechanisms of neutrophil-mediated defense against Hia that extend to both non-invasive and invasive strains.

**Conclusions:**

Hia clinical isolates with diverse pathogenicity illustrated contrasting susceptibility to killing by immune mechanisms while maintaining the same capacity to activate neutrophil-like cells, further underscoring the need for additional studies on Hia's pathogenesis.

## INTRODUCTION

*Haemophilus influenzae* (Hi) is a human-restricted Gram-negative bacterial pathogen that causes a wide range of non-invasive or severe invasive infections including otitis media, meningitis, and pneumonia [1]. Hi strains are classified into 6 serotypes (types a-f based on their capsular antigens) or termed nontypeable (NTHi) if they lack a capsule [2]. Historically, serotype b (Hib) was the leading cause of bacterial meningitis in young children worldwide. The introduction of a vaccine in the late 1980s drastically decreased the incidence of invasive infection and respiratory carriage of Hib [3]. Through serotype replacement, non-type b strains now dominate the ecological niche previously occupied by Hib. Serotype a (Hia) has emerged as a significant cause of invasive disease in North American Indigenous populations [4, 5, 6].

The complement system kills bacteria via innate and adaptive immune mechanisms. It is critical in the defense against encapsulated bacteria that require opsonophagocytosis for destruction [7]. eutrophils are abundant innate immune cells that are indispensable in anti-bacterial defense [8]. Recent research has revealed their extensive functional plasticity, such as their ability to modulate the adaptive immune system [8, 9]. The immune system crosstalk occurs through cell-derived soluble factors and contact-dependent mechanisms, eg, via Fc receptors (FcRs) binding antibody-opsonized bacteria, followed by B-cell activation [9].

Despite extensive knowledge and studies on Hib, the immune response to Hia remains incompletely understood. The polysaccharide capsule, which protects bacteria from opsonophagocytosis, is the major virulence factor of Hi. The Hia capsule is similar in structure to Hib and likely aids in the immune evasion of both serotypes by a similar mechanism [6, 10]. Hi type a and b clinical isolates that vary in phenotypic and genotypic characteristics (such as encapsulation and sequence type) have been observed to have virulence-enhancing features [11, 12]. It is unknown if these characteristics contribute to advancing invasive rather than non-invasive disease, and there is still limited information on non-invasive Hia isolates [13, 14].

Considering that a combination of host defenses and pathogen virulence characteristics determine disease pathogenesis and outcomes, this study aimed to elucidate the role of neutrophils in the immune response to Hia and determine whether it depends on sequence type, capsule presence, and the ability to cause invasive or non-invasive disease. Hia clinical isolates of a dominant North American circulating sequence type (ST)-23, an ST-4 isolate with enhanced virulence that is genetically distinct from ST-23, and a representative nontypeable strain (NTHi 375) were used [15, 16]. An opsonophagocytosis assay (OPA) was used to evaluate Hia's susceptibility to killing and identify the role of neutrophils, complement, and antibodies in this process. An *in vitro* infection model assessed changes in surface molecule expression after Hia stimulation to clarify the role of neutrophils in mediating the response to invasive vs non-invasive Hia.

## METHODS

### HL-60 Cell Culture Conditions and Differentiation

The human myeloblastic leukemia cell line (HL-60) was stored in liquid nitrogen until thawed for culturing. Undifferentiated cells were maintained at a density between 1×10^5^ and 1×10^6^ viable cells/mL in RPMI 1640 medium (Sigma-Aldrich) supplemented with 20% heat-inactivated fetal bovine serum (FBS) (R&D Systems, Inc.), and 1% antibiotic-antimycotic (Life Technologies Corporation). Cells were incubated at 37ºC in 5% CO_2_ in T-25 flasks (Corning Incorporated), passaged every 3 to 4 days, and maintained up to passage 20. Cell count and viability were determined with a hemocytometer using a 1:1 dilution factor with 0.4% Trypan blue solution (GE Healthcare Bio-Sciences). To induce differentiation, established cultures at a density of 5–8×10^5^ cells/mL cells were diluted to 1×10^5^ cells/mL in RPMI 1640 medium supplemented with 20% heat-inactivated FBS, 1% antibiotic-antimycotic, and 1.25% dimethyl sulfoxide (DMSO) (Fisher BioReagents). Medium was replaced every 2 days; the cell concentration was kept below 1×10^6^ cells/mL, and cells reached differentiation after 9 days.

### Confirmation of Differentiation

Cell differentiation was confirmed by morphology (Supplementary Figure 1A) and with flow cytometry (Supplementary Figure 1B–C). Differentiation caused cell morphology to shift from large and round to small and irregular cells, which was consistent with the literature [17]. For flow cytometry analysis, approximately 0.5×10^6^ of undifferentiated or differentiated cells were harvested by centrifugation for 5 minutes at 500*g*. The cells were washed with ice-cold 1X phosphate-buffered saline (PBS) (Fisher BioReagents) supplemented with 10% FBS. Cells were immunostained with Alexa Fluor 488 fluorochrome-conjugated antibodies against a differentiation marker, CD11b, at a concentration of 0.1 μg/mL and incubated in the dark for 1 hour at 4ºC. Unstained differentiated cells served as a negative control to assess cell autofluorescence. Cells were washed 3 times with ice-cold 1X PBS with 10% FBS by centrifugation (400*g* for 5 minutes) and resuspended in 500 μL of PBS with 10% FBS. Cells were kept in the dark on ice until immediately before analysis. Flow cytometry analysis was done on the SONY SA3800 spectral cell analyzer with SA3800 Software (Sony Corporation), acquiring 10,000 total events.

**Haemophilus influenzae strains and bacterial culture conditions.** Clinical Hia isolates, ie, strains 11-139, 13-240, 14-61, 13-0074, and NTHi strain 375 were used (Table 1).

**Table 1.** *H. influenzae* Clinical Isolates Examined and Their Characteristics

Strain	Isolate	Sequence Type	Invasive	Encapsulation	Isolation source
Hia	11-139	23	Yes	Yes	Blood, adult [13, 15]
Hia	14-61	23	No	Yes	Middle ear, pediatric [15]
Hia	13-0074	23	Yes	No (mutated)	Blood, pediatric [Dr. Tsang: unpublished].
Hia	13-240	4	Yes	Yes	Blood, pediatric [15]
NTHi	375	N/A	No	No	Middle ear, pediatric [16]

Bacteria were taken from 1.5 mL frozen stock suspensions made in 750 μL brain heart infusion (BHI) (Teknova, Hollister) broth and 750 μL 50% glycerol (stored at −80ºC). Bacteria were grown for 16 hours on BHI plates (1.5% agar supplemented with 1 μg/mL nicotine adenine dinucleotide (NAD) and 10 μg/mL hemin at 37ºC and 5% CO_2_) Isolated colonies were transferred to 3 mL of growth factor-supplemented BHI broth, and the optical density at 600 nm (OD_600_) was determined using a spectrophotometer. The bacterial suspension was adjusted to 0.1 OD_600_, and 500 μL of the 0.1 OD_600_ bacterial suspension was added to 10 mL of fresh growth factor-supplemented BHI broth. The bacteria were grown to log phase (5–6 hours) in a 37ºC incubator with shaking at 125 rpm. Log-phase bacteria were harvested, and the suspension was adjusted to 0.1 OD_600_. Bacteria were diluted to the desired concentration using concentrations at 0.1 OD_600_ calculated from previously established growth curves.

### Opsonophagocytosis Assay (OPA)

The methods used for the OPA were based on those described by Winter and Barenkamp [18]. The dHL-60 cells were harvested by centrifugation (150*g*, 8 minutes, room temperature), resus-pended in growth medium, and cell number and viability were assessed. The desired number of cells were centrifuged under the same conditions. The supernatant was removed, and cells were resuspended in Hanks' buffer without Ca^2+^ and Mg^2+^ at 5 mL per 50 mL of centrifuged cell culture and kept at 37ºC in a 5% CO_2_ atmosphere until immediately before use.

Mid-log phase bacteria at 0.1 OD_600_ were used to create a working solution of 2.5×10^5^ colony forming units (CFU)/mL in opsonophagocytosis buffer (OB), ie, Veronal-buffered saline (Lonza) with 0.5% bovine serum albumin (BSA) (Sigma-Aldrich), 0.15 mM CaCl_2_, and 0.5 mM MgCl_2_. Bacteria were washed twice with OB and maintained at room temperature for <1 hour before use.

Pooled human serum was used as the serum source. It was created from the serum samples of 25 healthy adults from the area surrounding Thunder Bay, Ontario, Canada as previously described [13]. The serum was stored at −80°C, thawed to room temperature, and heat-inactivated by submerging the vial in a 56°C water bath for 30 minutes. Heat-inactivated serum was serially diluted (2-fold) in 50 μL of OB until a dilution of 1:16 was reached. After the serum was diluted, 10 μL of each dilution was added to a microtiter plate. Then, 20 μL of bacterial suspension (approx. 5×10^3^ CFU) was added to each well with diluted serum. The plate was incubated at 37°C in 5% CO_2_ for 15 minutes. During the incubation period, dHL-60 cells were centrifuged (150*g*, 8 minutes, room temperature), the supernatant discarded, and the pellet gently resuspended in 2 mL of OB. Following the incubation period, 15 μL of 3-week-old baby rabbit serum (Pel-Freeze) as a complement source was added to each well.

Immediately after, 60 μL of dHL-60 cells (approx. 5×10^4^ cells) were added to each well to establish an effector-to-target cell ratio of 10:1 and ensure maximum phagocytosis (no differences were seen with a higher ratio [100:1 or 400:1]) [18]. The plate was incubated at 37ºC for 90 minutes with horizontal shaking (220 rpm). Subsequently, 10 μL aliquots from each well were plated onto supplemented BHI agar plates with multiple replicates. Plates were incubated for 16 hours at 37ºC in 5% CO_2_, and bacterial CFU were counted.

For each treatment, the desired component was replaced with the nutrient equivalent non-immunogenic component (Heat-inactivated rabbit complement replaced serum/complement and buffer replaced dHL-60 cells). Treatments included: Complete OPA (Hia + dHL-60 + Complement + Serum), Effector cell control (Hia – dHL-60 + Complement + Serum), Complement control (Hia + dHL-60 – Complement + Serum), Serum control (Hia + dHL-60 + Complement – Serum), Negative control (Hia). Results were reported as percent killing: [(CFU_Neg control_ - CFU_Treatment_)/CFU_Neg control_] × 100%.

### *In vitro* Model of Infection

The methods used were developed based on previous studies in our lab and published *in vitro* studies [19]. The dHL-60 cells were harvested by centrifugation (500*g*, 5 minutes, room temperature) and resuspended in RPMI with 20% FBS. Cell number and viability were assessed before use. Using the previously prepared 0.1 OD_600_ bacterial suspensions, the desired number of bacteria (MOI 1 or 100) was washed twice with PBS and resuspended in 200 μL of PBS. This new suspension was added to the wells containing 5×10^5^ dHL-60 cells and incubated for 1 hour at 37ºC, 5% CO_2_. After 1 hour, bacteria were killed with 220 μL of 1 mg/mL gentamicin prepared in sterile water (final concentration 100 μg/mL). The plate was incubated for an additional 71 hours. Bacterial killing was confirmed by plating 10 μL aliquots from each well onto supplemented BHI agar plates and observing growth overnight. Positive control cells were incubated with 100 ng/mL or 1 μg/mL of a potent immune activator, *Escherichia coli* (*E. coli*) LPS (Invitrogen).

### Flow Cytometry Analysis of the Surface Molecule Expression on dHL-60 Cells

Following a 72-hour stimulation, the plate was placed on ice for 3 minutes. The cells were harvested by centrifugation and washed with ice-cold 1X PBS. Cell pellets were resuspended in PBS supplemented with 10% FBS and stained with 1 μg/mL phycoerythrin (PE)-conjugated mouse-antihuman ICAM-1 (BD Biosciences) or CD64 (Invitrogen) antibody and/or 1 μg/mL fluorescein isothiocyanate (FITC)-conjugated mouse-antihuman CD16 (Invitrogen) or CD89 (Invitrogen) antibody for 1 hour at 4ºC in the dark. Immediately after, the cells were washed 3 times with 1X PBS and analyzed using flow cytometry as described above. The desired population was gated based on light scattering properties and 10,000 gated events were collected. An unstained sample excluded auto-fluorescent cells from gating using SONY's automatic autofluorescence finder tool. A PE- or FITC-conjugated isotype control (Invitrogen) excluded non-specific antibody binding from the analysis. Cell death was measured by adding 1 μg/mL propidium iodide (PI) to each sample immediately before analysis. Mean fluorescence intensity (MFI) of the entire cell population was recorded.

### Statistics

Statistical differences were determined using SPSS (IBM). Data were a representation of at least 3 independent experiments, with the statistical test used specified in the figure legend. Linear regression analysis examined the dependency of percentage killing on serum dilution. For analysis of differences in percentage killing between isolates and molecule expression between treatments, a one-way ANOVA with Tukey post-hoc test or an independent sample *t*-test was used.

## RESULTS

### Susceptibility of Hia to Killing Depends on Sequence Type, Encapsulation, and Ability to Cause Invasive Disease

**Figure 1.**
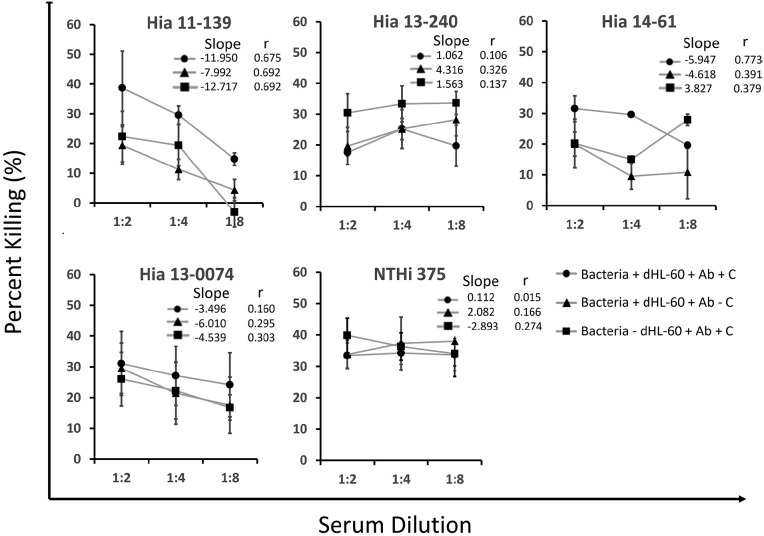
**Percentage killing of Hia strains in OPA over various serum dilutions.** Bacteria were incubated with pooled serum, rabbit complement, and dHL-60 cells, and 10 μL aliquots were plated and incubated overnight at 37ºC in 5% CO_2_. Percentage killing is displayed as the mean of multiple independent trials (n=3) ± standard error of the mean. The legend indicates the reaction mixture components. Ab = antibody and C = complement. Slope and Pearson r from linear regression analysis are shown.

The susceptibility of each isolate to killing was examined using OPA. The killing mechanisms of invasive vs non-invasive Hia strains were distinct, although the isolates had similar sensitivities to killing (killing ranged from 14.75% to 38.66% and 19.63% to 31.52% for Hia 11-139 and 14-61, respectively) (Figure 1). For each isolate, as serum (antibody source) was diluted, the percentage killing decreased. Removal of the individual components revealed differences in sensitivity to immune components. Both isolates displayed the lowest percentage killing when the complement was removed. Uniquely, the killing of Hia 11-139 constantly remained strongly correlated to serum dilution (r=0.692) when components were removed, and little to no killing was observed at the highest serum dilution, suggesting killing was heavily mediated by antibody opsonization.

Like Hia 11-139, killing decreased when complement or dHL-60 cells were removed. In contrast, while the percent killing of Hia 14-61 was originally strongly correlated (r=0.772) to serum dilution, correlation was weak when either complement (r=0.391) or dHL-60 cells (r=0.379) were removed, suggesting alternative effective destruction mechanisms could occur without antibodies (Figure 1). This difference was most apparent when dHL-60 cells were removed, in which Hia 14-61 had significantly higher killing than 11-139 at serum dilution 3 (*P*=0.007).

There was no correlation of killing with serum dilution for the unencapsulated strains Hia 13-0074 and NTHi 375. The killing of Hia 13-240 (ST-4) was also consistently weakly correlated to serum dilution, suggesting that serum did not contribute to killing, unlike ST-23 strains (Figure 1).

**Figure 2.**
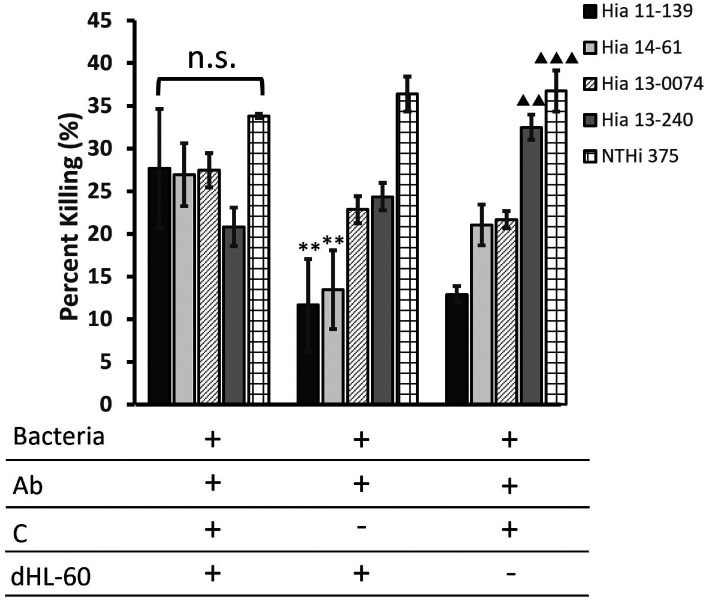
**Average percentage killing of Hia strains after exposure to multiple immunogenic components in an OPA.** Bacteria were incubated with pooled serum, rabbit complement, and dHL-60 cells, and 10 μL aliquots were plated and incubated overnight at 37ºC in 5% CO_2_. Percentage killing is displayed as the average over 3 serum dilutions of multiple independent trials (n=3) ± standard error of the mean. The legend indicates the reaction mixture components, in which Ab = antibody and C = complement. **P<0.01 compared to NTHi 375. ▴▴▴P<0.001, ▴▴P<0.01 compared to Hia 11-139 (one-way ANOVA with Tukey post-hoc test).

As expected, NTHi 375 consistently had the highest percentage killing among all the strains (ranging from 33.47% to 33.69%) (Figure 2). The killing of unencapsulated Hia 13-0074 (ST-23) was originally comparable to other ST-23 Hia strains (ranging from 24.11 to 31.1%). Both NTHi 375 and Hia 13-0074 exhibited a high percentage killing despite the removal of immune components, unlike the ST-23 encapsulated isolates. In this case, the percentage killing of NTHi, but not Hia 13-0074, was significantly higher than Hia 11-139 and 14-61 (*P*<0.01). Interestingly, removing dHL-60 cells also resulted in a significantly higher killing of Hia 13-240 than Hia 11-139 (*P*<0.01) (Figure 2).

### In Response to Invasive and Non-invasive Hia, Neutrophil-like Cells Show Analogous Changes in the Expression of Immunologically Important Molecules

To identify factors that may determine different clinical presentations of Hia infection, 2 encapsulated strains (11-139 and 14-61) with different abilities to cause invasive disease were used. Following stimulation of dHL-60 cells, flow cytometry analysis quantified surface expression of co-stimulatory molecule ICAM-1 and FcRs CD89 (FcαRI), CD64 (FcγRI), and CD16 (FcγRIIIa).

**Figure 3.**
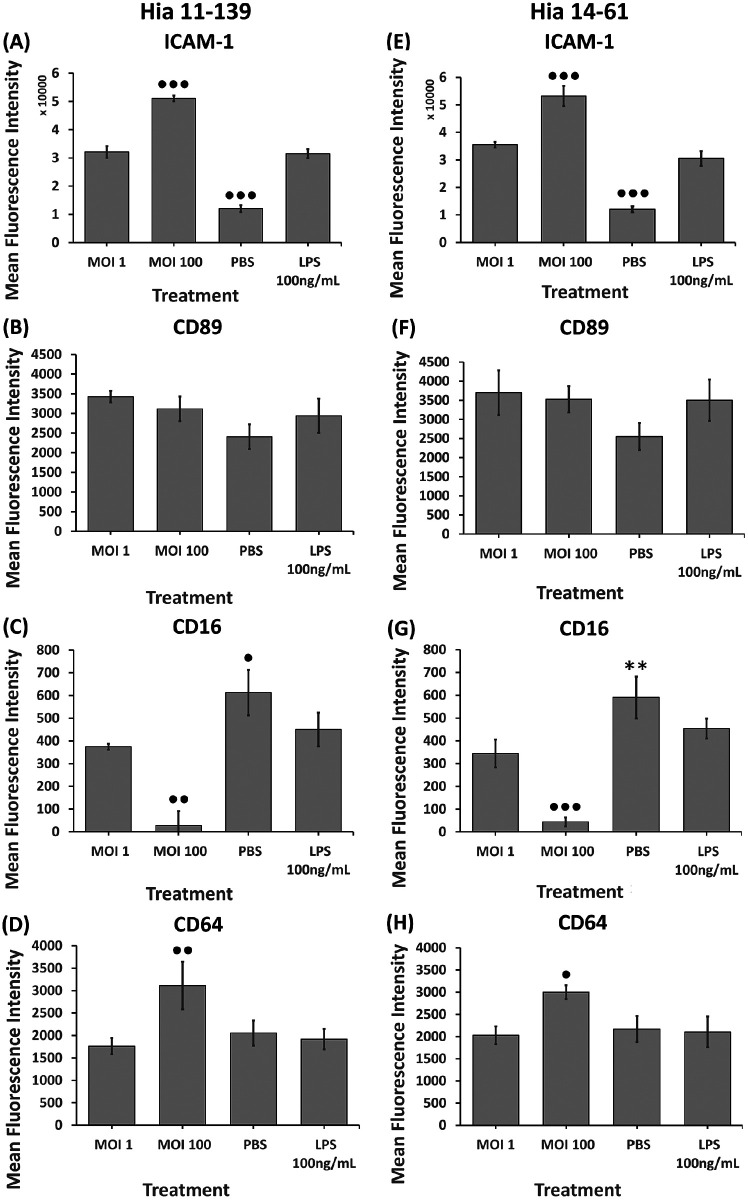
**Flow cytometry analysis of ICAM-1 (A, E), CD89 (B, F), CD16 (C, G), and CD64 (D, H) on dHL-60 cells after stimulation with Hia 11-139 (A-D) and 14-61 (E-H) at multiple MOIs.** Negative control = PBS, positive control = E. coli LPS. Harvested cells were stained with 1 μg/mL mouse-antihuman PE-conjugated ICAM-1/CD64 or FITC-conjugated CD89/CD16 antibody for 1 hour. Average MFI ± SD of all gated cells is shown, n ≥ 3. •••P<0.001, ••P<0.01, •P<0.05 compared to all other treatments, ✗✗P<0.01 compared to PBS, **P<0.01 compared to MOI 1, 100 (one-way ANOVA with a Tukey post-hoc test).

Both isolates induced a dose-dependent increase in ICAM-1 expression compared to the un-stimulated control (*P*<0.001) (Figure 3A,E). Hia 11-139 and 14-61 caused ICAM-1 expression to significantly increase between MOI 1 and 100 (*P*<0.001) (Figure 3A,E). Stimulation with Hia 11-139, 14-61, and LPS initially increased CD89 expression compared to the unstimulated control, although this did not reach significance (Figure 3B,F). CD89 expression decreased at MOI 100, which could be associated with lower cell viability at higher MOI (64.95% compared to 57.97% PI-negative cells for MOI 1 and 100, respectively) (data not shown). For CD16, stimulation with Hia 11-139, 14-61, and LPS induced a dose-dependent decrease in expression compared to the unstimulated control (*P*<0.05) (Figure 3C,G). Stimulation with MOI 100 resulted in a significant decrease in CD16 expression compared to all treatments (*P*<0.01). CD16 expression was negligible at MOI 100 (Figure 3C,G). Stimulation with Hia 11-139 and 14-61 caused a significant increase in CD64 expression at MOI 100 compared to all treatments (*P*<0.01 and *P*<0.05 for Hia 11-139 and 14-61, respectively) (Figure 3D,H). Stimulation with LPS or any bacterial dose lower than MOI 100 did not cause significant variation in CD64 expression compared to the unstimulated control (Figure 3D,H).

**Figure 4.**
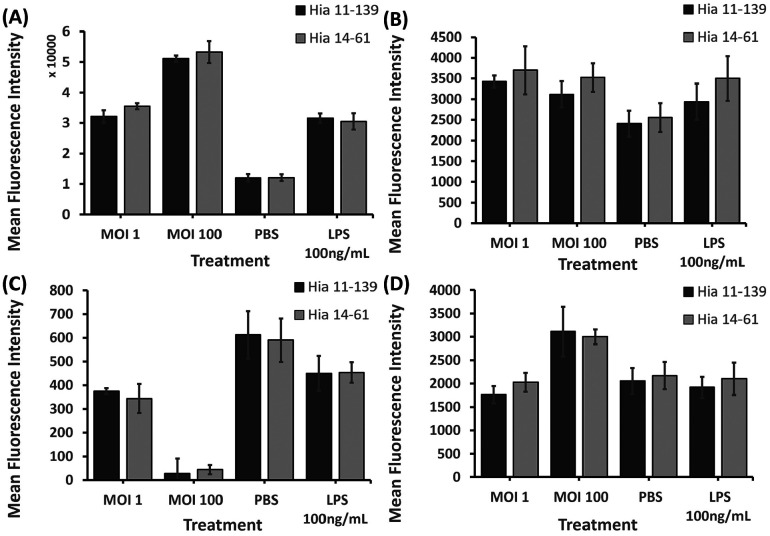
**Flow cytometry analysis of ICAM-1 (A), CD89 (B), CD16 (C), and CD64 (D) on dHL-60 cells after stimulation with Hia 11-139 and 14-61 (MOI 1, 100).** Negative control = PBS, positive control = E. coli LPS 100 ng/mL. Harvested cells were stained with 1 μg/mL mouse-antihuman PE-conjugated ICAM-1/CD64 or FITC-conjugated CD89/CD16 antibody for 1 hour. Average MFI ± SD of all gated cells is shown, n ≥ 3. *P<0.05 (independent samples t-test).

No significant differences were found in ICAM-1, CD89, CD16, and CD64 levels after stimulation with Hia 11-139 vs 14-61 (Figure 4). These findings suggest that invasive and non-invasive Hia isolates have the same capacity to alter dHL-60 cell surface receptor expression.

## DISCUSSION

Invasive *H. influenzae* disease remains an important concern despite the successful use of Hib-conjugate vaccines for pediatric immunization [4, 14]. In our study, an OPA and *in vitro* infection model evaluated neutrophil defense mechanisms against invasive and non-invasive Hia isolates. The results demonstrated that Hia isolates varied in their susceptibility to killing but not in their capacity to activate neutrophil-like cells.

OPAs have been successfully utilized with various bacterial species as a useful *in vitro* method of evaluating bacterial resistance to killing [17]. In this system, serum antibodies are present along with complement and neutrophils. Specific antibodies opsonize the pathogen, creating antigen-antibody complexes, followed by neutrophil FcRs and complement receptor engagement that will induce opsonophagocytosis and/or the classical complement pathway. Additionally, the alternative complement pathway is activated in response to bacteria independently of antibodies. Complement activation causes opsonization, membrane-attack complex (MAC) formation, and the release of inflammatory mediators [20]. Removing or adding particular components can help to identify strain-specific sensitivities to immune defense mechanisms.

The capsule is a critical virulence determinant that enhances bacterial survival [21]. As expected, unencapsulated isolates (Hia 13-0074 and NTHi 375) experienced greater killing than encapsulated isolates (Hia 11-139, 14-61, 13-240). The killing of the mutated unencapsulated strain Hia 13-0074 remained lower than NTHi 375, suggesting it retained some Hia virulence factors that contributed to its survival. Notably, the killing of NTHi 375 did not change with the addition or loss of any components. In the absence of a capsule, NTHi strains are constantly under selective pressure to develop resistance mechanisms against serum and complement, including exhibiting extensive heterogeneity in lipooligosaccharide glycoforms, binding complement-inhibitory proteins, and undergoing phase variation of the outer membrane proteins [22, 23].

The killing of the unencapsulated Hia 13-0074 and encapsulated Hia 13-240 showed minimal correlation to serum dilution. Anti-capsular antibodies and antibodies to cell wall components may have different capacities to kill Hi; indeed, previous observations revealed that NTHi 375 was moderately resistant to serum [24]. Hia 13-240 (ST-4) contain an IS*1016-bexA* partial deletion and are associated with severe disease and high case-fatality rate [12, 15]. The origin of naturally acquired specific antibodies against Hia is undetermined, and the sensitivity of individual clinical isolates to their bactericidal effect varies [25]. As ST-4 Hi do not circulate in our area, the pooled human serum from this region may lack protective antibodies against Hia 13-240. Although Nix et al found similar sensitivity of Hia 11-139 (ST-23) and 13-240 (ST-4) in the serum bactericidal assay (SBA) [25], lack of serum sensitivity of Hia 13-240 in OPA may be attributed to undefined virulence factors. Unexpectedly, Hia 13-240 experienced significantly higher killing than 11-139 when dHL-60 cells were removed. Hia 13-240 may have additional virulence factors that increase its survival in the presence of neutrophils. For example, many gram-negative bacteria are known to survive in neutrophils after phagocytosis, such as *Bordetella pertussis, Legionella pneumophila,* and *Haemophilus somnus* [26].

Though both Hia 11-139 (invasive) and Hia 14-61 (non-invasive) are encapsulated ST-23 strains, differences in their pathogenicity could be caused by variations in the amount of capsular material. For example, several invasive Hib isolates contain 5 or more copies of the *cap* locus, causing them to be heavily capsulated [11].

The killing of Hia 11-139 relied on opsonization by antibodies and complement, suggesting that invasive strains require classical complement activation for killing. This supports previous observations that opsonization with capsular and non-capsular antibodies was necessary to kill invasive Hi strains [13, 25]. By contrast, in the case of non-invasive Hia 14-61, bacterial survival did not depend on serum dilutions in the absence of dHL-60 cells, suggesting the role of the alternative complement pathway. This is consistent with previous findings that Hia 11-139 was more sensitive to the complement-dependent SBA than 14-61 [25]. In the presence of neutrophil-like cells, opsonophagocytosis of Hia 14-61 may depend on the engagement of complement receptors.

As a result of bacterial recognition, neutrophils become activated, which leads to alterations of the surface expression of certain molecules, followed by the initiation of intracellular signaling processes required for bacterial destruction [28]. We conducted additional analyses to ascertain if invasive and non-invasive strains caused comparable dHL-60 phenotypic alterations consistent with cell activation and inflammatory response, with a focus on the expression of ICAM-1 and several FcRs. ICAM-1 is an adhesion and co-stimulatory molecule responsible for regulating multiple immune responses, including inflammation, leukocyte recruitment, and T-cell stimulation. Reports on its expression and anti-bacterial function on neutrophils are scant [29]. Neutro-phil FcRs recognize antigen-antibody complexes, facilitating opsonophagocytosis and mediating inflammation [28].

Hia 11-139 and 14-61 upregulated ICAM-1, CD89, and CD64 and downregulated CD16 expression, with no strain-specific differences noted, suggesting both isolates induced a similar early-phase inflammatory response, and differences in their pathogenicity lay elsewhere. Nevertheless, the analysis identified potential mechanisms behind neutrophil effector function activation. We found ICAM-1 expression increased in a dose-dependent manner following Hia stimulation. Its increase has previously been associated with enhanced inflammation, reactive oxygen species (ROS) production, and phagocytosis through TLR4-dependent PI3K signaling on macrophages stimulated with LPS [30]. This suggests upregulation of ICAM-1 might contribute to the immune response to Hia by enhancing neutrophil effector functions, but the exact mechanisms remain to be understood.

Although the functional role of Fc[.alpha]RI/CD89 (FcR for IgA) in anti-bacterial defenses is incompletely understood, CD89 was found to regulate neutrophil survival [31]. Enhanced neutrophilic phagocytosis of several other bacteria was previously observed following CD89 activation [32]. In our study, CD89 expression increased at low MOIs, suggesting dHL-60 cells could acquire enhanced abilities to phagocytose Hia. However, CD89 expression decreased at higher MOI, suggesting cell death. Previous studies have observed neutrophil cell death following the generation and release of neutrophil extracellular traps (NETosis) after phagocytosis of IgA-opsonized bacteria via CD89 [33]. Indeed, our study found increased cell death with increasing Hia and LPS stimulation with PI-staining, but this hypothesis warrants further research (data not shown).

The expression of the low-affinity Fc[.gamma]RIIIa (CD16) decreased in a dose-dependent manner, corroborating earlier observations on LPS stimulation of human monocytes [34]. CD16 is co-expressed with a high-affinity FcγRII (CD32A), and it regulates cellular activation following receptor engagement by antigen-antibody complexes [35]. In resting neutrophils, CD16 extends out further from the cell membrane than CD32A, making it more likely to capture antigen-antibody complexes, hence preventing CD32A activation that could lead to strong pro-inflammatory signaling [35]. Our results suggest the shedding of CD16, which could be followed by an increased CD32A engagement. This would result in neutrophil activation via CD32A-mediated signaling, potentially allowing for increased effector functions.

Increased expression of the high-affinity FcγRI (CD64) occurred following incubation of dHL-60 cells with Hia at MOI 100 but not at MOI 1, suggesting that a low bacterial stimulation was insufficient to activate neutrophil-like cells to a degree needed to produce this receptor. Yet, at a higher bacterial load, the engagement of FcγRI could augment neutrophil functional abilities by enhancing phagocytosis, ROS production, or cytokine release via the activation of ITAM-mediated signaling pathways [36, 37]. Considering that in some clinical studies, increased CD64 expression during sepsis was interpreted as an indicator of abnormal neutrophil activity, the functional role of CD64 in Hia infections deserves further study [35, 36].

This study was limited by its use of dHL-60 cells. The results must be confirmed using primary cells or an *in vivo* model, as dHL-60 cells do not express all neutrophil receptors and lack some neutrophil granules [38]. This study provided evidence of opsonophagocytosis and neutrophil receptor engagement in an *in vitro* infection model, although accurate mechanisms of neutrophil defenses against Hia remain to be fully understood. This is the first characterization of the effects of North American clinical Hia isolates with diverse phenotypes/genotypes and pathogenic potential. However, this study should be applied to other Hia strains to develop a deeper knowledge of Hia's pathogenesis. Due to high genetic variability in NTHi strains, these findings cannot be generalized to all NTHi.

## CONCLUSION

Our study results contribute to understanding of the mechanisms of host defense against Hia. This is important considering the significant burden of invasive Hia disease in North American Indigenous populations. In certain geographic areas, ie, the North American Arctic, Hia disease incidence rates among children are now approaching those of invasive Hib in the pre-Hib vaccine era [39]. We have found that clinical Hia isolates exhibit different susceptibility to host defense mechanisms depending on their characteristics and source of isolation. In particular, encapsulated Hia had an increased resistance to opsonophagocytosis compared to unencapsulated Hi. Moreover, antibody-mediated opsonization was required for killing invasive but not non-invasive Hia. Yet, invasive and non-invasive isolates had similar abilities to activate neutrophil-like cells, suggesting that the development of invasive disease is likely associated with a lack of antibody-mediated opsonization rather than the impairment of the functional capabilities of the neutrophils.

Altogether, our findings emphasize the significance of antibody-mediated opsonization in defense against Hia causing invasive disease. Indeed, naturally acquired serum antibodies collected from an adult population where ST-23 strains circulate were indispensable for neutrophilic killing capacity towards an invasive ST-23 isolate (Hia 11-139). Of note, this isolate was used to prepare an antigen included in a new polysaccharide-protein conjugate Hia vaccine recently developed in Canada which enters the phase 1 clinical trial in 2024 [25]. Pediatric immunization with this vaccine may protect vulnerable populations against invasive disease via inducing Hia-specific antibodies in children who are too young to produce sufficient amounts of naturally acquired antibodies for opsonization.

## SUPPLEMENTARY DATA

Supplementary materials are available at the *Pathogens and Immunity* website. Supplementary data may be provided by the authors to benefit the reader. Supplementary data are not copyedited and are the sole responsibility of the authors. Questions or comments related to supplementary materials should be addressed to the corresponding author.
