# Supplementary material for: Invasive and Non-invasive Clinical Haemophilus influenzae Type A Isolates Activate Differentiated HL-60 Cells In Vitro
Source: Pathog Immun. 2024 Apr 17;9(1):38–55. doi: 10.20411/pai.v9i1.659 (PMC11107419; doi:10.20411/pai.v9i1.659)
Supplement: Supplementary Figure 1 [file pai-9-038-s01.pdf]

PATHOGENS AND IMMUNITY
In Association with Case Western Reserve University and The Richard J. Fasenmyer Foundation

SUPPLEMENTARY FIGURE 1

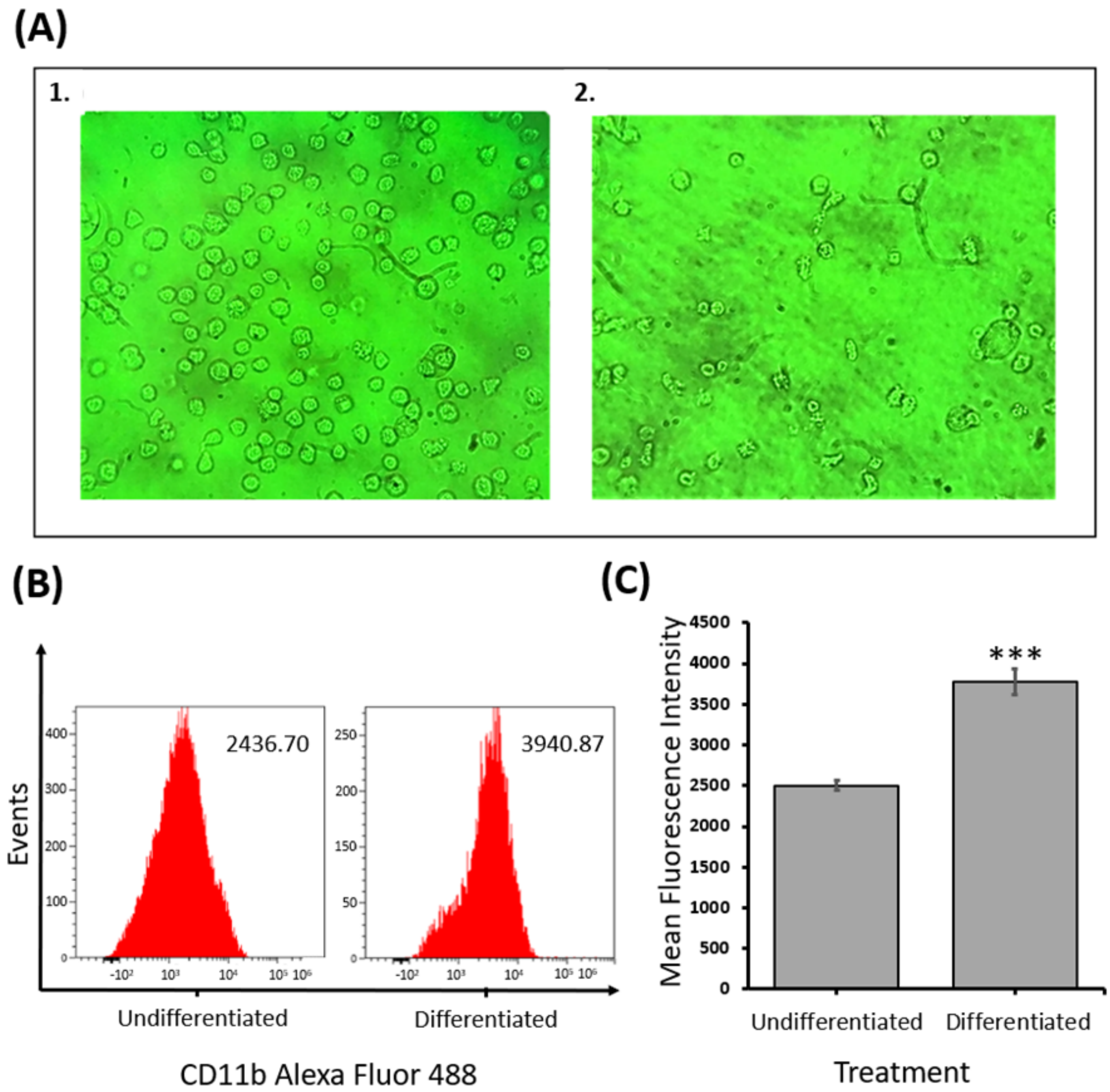

Supplementary Figure 1. Confirmation of differentiation using morphology (A) and flow cytometry (B-C). HL-60 cells were induced to differentiate into neutrophil-like cells over 9 days using 1.25% DMSO in growth medium. Undifferentiated cells were grown without DMSO and split every 2-3 days. Cells (1×10^6) were stained with 0.1 µg/mL Alexa Fluor 488 fluorochrome-conjugated primary antibodies against CD11b. (A): Microscopic view (40X) of HL-60 cells before (1) and 9 days after (2) DMSO incubation. (B) Histograms of one representative experiment showing CD11b expression (MFI) in undifferentiated and differentiated cells (C) Data are expressed as the MFI ± SD (n=3) ($p<0.001$).

Ferris CL, Ulanova M. Invasive and Non-invasive Clinical *Haemophilus influenzae* Type A Isolates Activate Differentiated HL-60 Cells *In Vitro*. *Pathogens and Immunity*. 2024;9(1):38–55. doi: 10.20411/pai.v9i1.659
